# End-to-end learning for compound activity prediction based on binding pocket information

**DOI:** 10.1186/s12859-021-04440-w

**Published:** 2021-10-29

**Authors:** Toshitaka Tanebe, Takashi Ishida

**Affiliations:** Department of Computer Science, School of Computing, Tokyo Institute of Technology, 2-12-1 W8-85 Ookayama, Meguro-ku, Tokyo, 152-8550 Japan

**Keywords:** Drug discovery, Virtual screening, Docking simulation, Graph convolution, Deep neural network

## Abstract

**Background:**

Recently, machine learning-based ligand activity prediction methods have been greatly improved. However, if known active compounds of a target protein are unavailable, the machine learning-based method cannot be applied. In such cases, docking simulation is generally applied because it only requires a tertiary structure of the target protein. However, the conformation search and the evaluation of binding energy of docking simulation are computationally heavy and thus docking simulation needs huge computational resources. Thus, if we can apply a machine learning-based activity prediction method for a novel target protein, such methods would be highly useful. Recently, Tsubaki et al. proposed an end-to-end learning method to predict the activity of compounds for novel target proteins. However, the prediction accuracy of the method was still insufficient because it only used amino acid sequence information of a protein as the input.

**Results:**

In this research, we proposed an end-to-end learning-based compound activity prediction using structure information of a binding pocket of a target protein. The proposed method learns the important features by end-to-end learning using a graph neural network both for a compound structure and a protein binding pocket structure. As a result of the evaluation experiments, the proposed method has shown higher accuracy than an existing method using amino acid sequence information.

**Conclusions:**

The proposed method achieved equivalent accuracy to docking simulation using AutoDock Vina with much shorter computing time. This indicated that a machine learning-based approach would be promising even for novel target proteins in activity prediction.

## Background

These days, A typical drug discovery process takes 12–14 years and costs about $ 2.6 billion [1, 2]. As one of the solutions to this problem, computer-aided drug discovery (CADD) has become one of the most efficient methods to reduce these costs. At the initial stage of drug discovery, a compound screening is often performed for selecting drug candidate compounds from a chemical compound library. Such a library sometimes contains millions to tens of millions of compounds and the cost of the screening cannot be ignored. Virtual screening is a computational method to predict the activity of chemical compounds without biochemical experiments. The accuracy has been improved recently and the technology has been applied successfully [3].

The virtual screening technique can be roughly divided into two categories: ligand-based virtual screening (LBVS) methods and structure-based virtual screening (SBVS) methods. LBVS predicts the activity of a given compound only from its chemical structure and supervised machine learning algorithms are used for the prediction. The method generally shows a good performance if we have a sufficient number of activity data for a target protein. However, LBVS cannot be applied if we have no compound activity data of a target protein. In contrast, SBVS can be used even if there is no activity information of a target protein. SBVS generally performs a simulation based on physicochemistry using the 3-dimensional structure of a target protein for predicting the activity of a compound. Docking simulation is one of the most major SBVS methods. It predicts the binding energy of a compound to a target protein by virtually docking the compound (i.e., ligand) with a binding site (i.e., pocket) of a target protein surface.

Currently, various docking simulation software, such as AutoDock Vina [4], Glide [5], and eHiTS [6], have been developed and widely used in practical research projects [7]. However, docking simulation has a problem. Docking simulation needs to search a large conformational space by rotating and translating a compound and calculate the binding energy of a protein-compound interaction using a complicated score function. This process is computationally expensive and takes a few minutes to predict the activity of one compound with a single CPU core [5]. The execution time of docking simulation of a compound is acceptable. However, for virtual screening, we have to perform the process for a large number of compounds. Therefore, even if a docking simulation can evaluate one compound in 10 s, a screening of a compound library includin10 million compounds requires approximately 1200 CPU days.

In order to tackle this problem, several researchers proposed machine learning-based virtual screening methods to predict the activity of a compound for a novel target protein. Such researches used not only a chemical compound structure but also protein information as the input of the machine learning models. Recently, Tsubaki et al. proposed an end-to-end learning prediction method for the problem and achieved better accuracy than the previous methods [8]. End-to-end learning combines feature design and task learning at the same time. By directly learning the relationship between the input and the output, it is possible to obtain a better representation of the input data rather than to manually encode it. The accuracies of end-to-end learning-based methods were often higher than those of conventional methods in various applications, such as Diagnosis of X-ray Images [9]. In the research of Tsubaki et al., they used a graph neural network for a compound and a one-dimensional convolutional neural network for a protein amino acid sequence. They managed to avoid manual encoding of input vectors. However, this method has some room for improvement because the method used only amino acid information. Structure information of a protein, especially the pocket structure, is considered to contain more useful information for ligand binding prediction. As illustrated by the key and keyhole theory, a binding compound often has a specific shape for the pocket structure of a target protein. Thus, the pocket structure information is more important for binding estimation than the amino acid sequence which only implies the pocket information. In addition, when using an amino acid sequence, it is assumed that there is sequence homology between a target protein and a protein in the training data set. However, using a pocket structure, it may be possible to predict the activity even for a novel target protein without any sequence homology.

In this paper, we proposed a method to predict the activity of a compound by end-to-end learning using the pocket structure information of a protein. The proposed method uses graph neural networks both for compound and protein pocket structures. As a result of the benchmark on a data set for virtual screening performance evaluation, the proposed method achieved higher accuracy compared to the previous method which uses amino acid sequence information as the input.

## Results

### Dataset

We used the DUD-E (a Database of Useful Decoys: Enhanced) dataset [10] as a training dataset of machine learning-based prediction models. DUD-E is a dataset constructed for the performance evaluation of the structure-based screening method created by Mysinger et al. A total of 102 target proteins were selected considering diversity, and active compounds and decoy compounds were prepared for each target. In total, the dataset contains 22,886 active compounds and more than 1 million decoy compounds. In this study, a down-sampled version at a 1:1 ratio of active to decoy was used as the training dataset. We checked that proteins in the training dataset had no sequence similarity to those in the test dataset using NCBI-BLAST [11] as described below (the sequence identity was lower than 30%.)

We used the MUV (Maximum Unbiased Validation) dataset [12] as our test dataset because the three-dimensional structure of the target protein is required. Rohrer et al. obtained the assay data for 17 target proteins from the bioactivity data contained in PubChem [13], and assigned 30 active compounds and 15,000 decoy compounds to each target protein. In this study, a total of 9 target proteins, whose protein–ligand complex structure has been solved, was used. Those proteins are described in Table 1. This is the same dataset as used in the research by Ragoza et al. [14]

**Table 1:** Details of the selected MUV dataset

MUV ID	PDB ID	Description	Ligand	Assay type
600	1yow	Steroidogenic factor 1: inhibitors	P0E	Cell
692	1yow	Steroidogenic factor 1: agonists	P0E	Cell
859	5cxv	Muscarinic receptor M1	0HK	Cell
852	4xe4	Factor XIIa	NAG	Biochemical
548	3poo	Protein kinase A	S69	Biochemical
832	1au8	Cathepsin G	0H8	Biochemical
689	2y6o	Ephrin receptor A4	1N1	Biochemical
846	5exm	Factor XIa	5ST	Biochemical
466	3v2y	Sphingosine 1-phosphate receptor	ML5	Cell

### Evaluation measure

We used AUROC (Area Under Receiver Operating Characteristic) as an evaluation index. AUROC is an index using the area under ROC curve, and it is an evaluation index mainly used for binary classification problems. ROC is a curve with a true positive rate (TPR) on the vertical axis and a false positive rate (FPR) on the horizontal axis. TPR is the proportion of positives correctly identified as positive in the dataset, and FPR is the proportion of negatives incorrectly identified as positive in the dataset. TPR and FPR can be calculated by the following formulas.

$$\begin{array}{*{20}c} {TPR = \frac{\# TP}{{\# TP + \# FN}}} \\ \end{array}$$

$$\begin{array}{*{20}c} {FPR = \frac{\# FP}{{\# FP + \# TN}}} \\ \end{array}$$

We also used F1-score for the evaluation. F1-score is the harmonic mean of the precision and recall. The precision is the number of correctly identified positive results divided by the number of all positive results, including those not identified correctly. The recall is the number of correctly identified positive results divided by the number of all samples that should have been identified as positive.

### Prediction accuracy evaluation

For checking the improvement of the prediction accuracy of the proposed method, we performed the evaluation on the MUV dataset. We compared the prediction accuracy of the proposed method with the method using sequence information by Tsubaki et al. and docking simulation using AutoDock Vina, which is one of the popular docking simulation software.

Table 2 shows the results of AUROC. The proposed method achieved better accuracy than both, Tsubaki et al. and AutoDock Vina. However, the improvement of the proposed method compared to Tsubaki et al.’s method was not that big. As shown in Table 2, the proposed method has shown better accuracy for almost all targets (8/9 targets). In addition, judging from the results in Table 2, the proposed method has shown the best accuracy for 6 out of the total 9 target protein. To check the statistical significance of the improvement by the proposed method, we performed a paired t-test at the 5% level of significance. The p-values of the test between the proposed method and Tsubaki et al. and AutoDock Vina were 0.039 and 0.149, respectively. Thus, the improvement of the proposed method was significant compared to Tsubaki et al., but compared to AutoDock Vina the improvement was not significant because of its high variance.

**Table 2:** Prediction performance for selected MUV dataset (AUROC)

MUV ID	Pocket structure (Proposed)	Sequence (Tsubaki et al.)	Docking (AutoDock Vina)
600	0.574	0.539	0.555
692	0.542	0.531	0.470
859	0.508	0.498	0.509
852	0.647	0.643	0.482
548	0.721	0.707	0.482
832	0.612	0.599	0.535
689	0.467	0.481	0.547
846	0.631	0.630	0.461
466	0.409	0.404	0.613
Average	0.568	0.559	0.517

Table 3 shows the results of the F1-score evaluation. The proposed method has shown better accuracy in comparison to Tsubaki et al. and AutoDock Vina as well as in AUROC. We also checked the statistical significance of the improvement for the F1-score. The p-value of the improvements for Tsubaki et al. and AutoDock Vina were 0.047 and 0.051, respectively. For the F1-score, the difference between the accuracy of the proposed method and that of AutoDock Vina was clearer than in AUROC, but the improvement was also not significant.

**Table 3:** Prediction performance for selected MUV dataset (F1-score)

MUV ID	Pocket structure (Proposed)	Sequence (Tsubaki et al.)	Docking (AutoDock Vina)
600	0.138	0.029	0.031
692	0.031	0.026	0.014
859	0.041	0.019	0.023
852	0.241	0.199	0.011
548	0.329	0.263	0.020
832	0.122	0.131	0.041
689	0.011	0.021	0.051
846	0.112	0.112	0.009
466	0.010	0.006	0.117
Average	0.115	0.090	0.035

## Discussion

### Evaluation of computing time

The proposed method achieved to improve overall prediction accuracy. However, such methods are not so useful if they require significant computing resources. Therefore, we also evaluated the computing time. The prediction time was measured on an f-node of supercomputer TSUBAME3.0 at Tokyo institute of Technology. The details are shown in Table 4. AutoDock Vina used a single CPU core. The proposed method and Tsubaki et al.’s method used a single CPU core and a GPU card. The results of the prediction time per compound are shown in Table 5. The proposed method took a long time to complete the prediction compared to Tsubaki et al. because the pocket graph used in the proposed method is more complicated than the 1-dimensional convolution neural network used in that research. However, compared with AutoDock Vina, the prediction time is much faster (more than 1000-fold acceleration), and we think that the performance is still sufficient to be useful for practical usage.

**Table 4:** Details of the computing environment

CPU	Intel Xeon E5-2680 v4 2.4 GHz × 2
Number of CPU core	28 core
Memory	240 GB
GPU	NVIDIA TESLA P100 for NVlink-Optimized Servers × 4

**Table 5:** Requied prediction time per a single compound

Pocket structure (Proposed)	Sequence (Tsubaki et al.)	Docking (AutoDock Vina)
0.011 [s]	0.0034 [s]	14.37 [s]

## Conclusions

In this research, we proposed a new end-to-end learning method for activity prediction using protein pocket structure information. The proposed method has shown higher prediction accuracy than previous methods. Furthermore, compared with docking simulation software, the proposed method has shown higher accuracy in a much shorter computing time. Unfortunately, the improvement of the proposed method was not statistically significant in the case of AutoDock Vina. One clear reason why there was no statistical significance is the small size of the dataset used in the evaluation. In this research, we used only 9 protein targets for the evaluation, which clearly is not enough for this type of evaluation. However, besides the MUV dataset, there is currently no available dataset that is sufficiently unbiased. For instance, the DUD-E dataset, which was used in previous research, has a bias and it is not suitable for the comparison between docking and machine learning-based methods [15]. Thus, we used it only as the training dataset and evaluated the performance based on a subset of the MUV dataset. The development of a larger dataset that is more suitable for our approach is one of our future works. In this research, the proposed method has shown worse accuracy than AutoDock Vina for 2 proteins (MUV ID 689 and 466). Unfortunately, we could not find any clear reason for that, but we will be able to analyze such things based on a larger dataset.

In addition to a new dataset construction, currently, we use only protein pocket structure information as a protein feature, but a combination of amino acid information and protein pocket structure information may improve overall prediction accuracy.

## Materials and methods

The proposed method uses pocket structure information instead of amino acid sequence information used in the previous method as the feature of a protein and applies a graph neural network for not only a compound but also a protein. The generation of compound features is the same as the previous method by Tsubaki et al. [8], but protein feature generation is highly different. The proposed method consists of the following three parts; (1) Graph generation of a compound structure and a protein pocket structure. (2) Feature learning by graph neural network. (3) Activity prediction by a classifier.

### Graph generation of a compound structure and protein pocket structure

A compound structure was firstly converted from a SMILES format string into a graph structure consisting of vertices (atom types) and edges (chemical bonds) by RDKit. If a SMILES-formatted file contains dots (represents non-concatenation), it is excluded from the data set because the generation of a single graph is not possible.

**Fig. 1:**
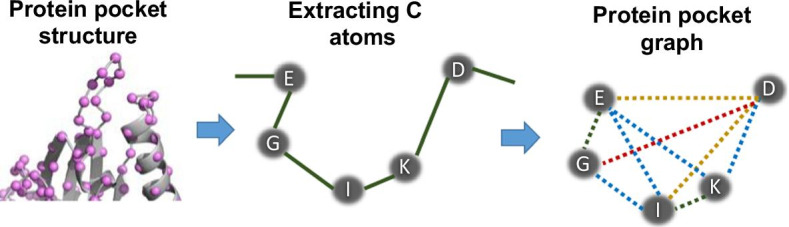
Conversion of a protein pocket structure into a graph. Each circle is a residue (vertex). Green line, blue line, yellow line, red line, and gray line mean type I, II, III, IV, and V edges, respectively

Protein pocket structure information was extracted as the information (residue type, coordinates) of ligand contact residues identified by LPC software [16]. Then the information was converted into a graph. The vertices of the graph correspond to each residue. The edges of the graph correspond to residue interactions including bonded and non-bonded ones (Fig. 1). The vertices were categorized by their amino acid type (20 types) and represented as 20-dimensional one-hot vectors. The edges were categorized into five types according to the distance between Cα atoms of residues (I: 1.0–4.8 Å, II: 4.8–7.0 Å, III: 7.0–9.2 Å, IV: 9.2–11.4 Å, V: 11.4–13.6 Å). The types of edges were defined according to previous related research by Ito et al. [17]. By roughly grouping the distance between Cα atoms, we expected that it can cope with the structural change of a protein pocket.

### Graph neural network

**Fig. 2:**
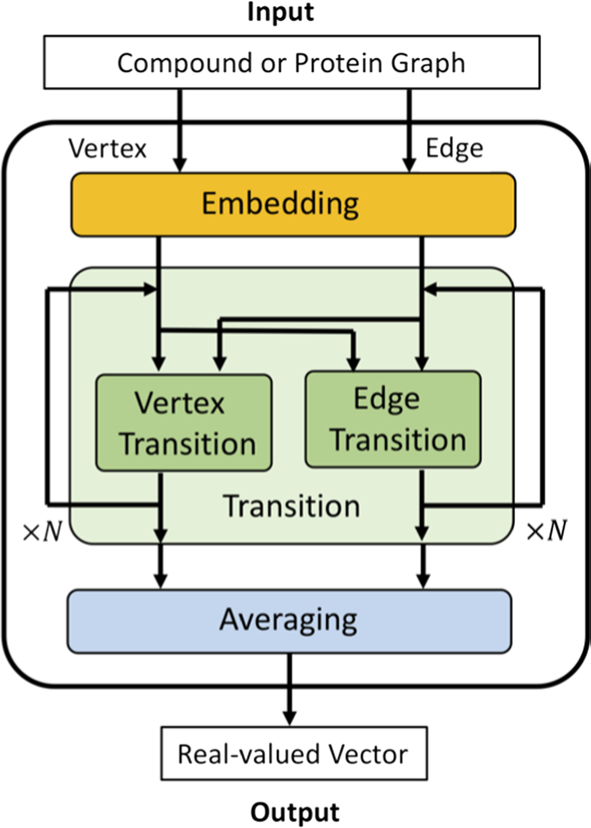
Representation learning by graph neural network

The compound and protein pocket graphs generated by the procedure described above are each converted to real-valued vectors by using a graph neural network. The procedure of the graph neural network consists of three parts (embedding, transition, and averaging) both for compound graphs and protein pocket graphs (Fig. 2).

In the embedding part, we employed the same method used in research by Tsubaki et al. [8]. We defined *r*-radius vertex and *r*-radius edge, which introduced the concept of *r*-radius subgraphs [18] to vertex and edge, respectively. The *r*-radius subgraphs include all neighboring vertices and edges within a radius *r* (*r* is the number of hops on the graph) from a certain vertex. In the transition part, the following operation (i) and (ii) for each *r*-radius vertex and each *r*-radius edge are repeated. (i) Add adjacent r-radius vertex and r-radius edge vector. (ii) The vector generated by the operation (i) is input to the non-linear function and updated. Finally, all *r*-radius vertex vectors generated by the operation (ii) are averaged and one real-valued vector is output in the averaging part.

### Activity prediction by a classifier

The activity of a compound was predicted using the *d*-dimensional compound vector ${\varvec{y}}_{molecule}$ and the protein vector ${\varvec{y}}_{protein}$ obtained from a graph neural network described above. Firstly, we simply concatenated ${\varvec{y}}_{molecule}$ and ${\varvec{y}}_{protein}$ as follows: [${\varvec{y}}_{molecule} ;\user2{ }y_{protein} ]$. Then, the input ${\varvec{z}} \in {\mathbb{R}}^{2}$ to a softmax layer is obtained by the following equation:

$$\begin{array}{*{20}c} {z = {\varvec{W}}_{output} \left[ {{\varvec{y}}_{molecule} ;{\varvec{y}}_{protein} } \right] + {\varvec{b}}_{output} } \\ \end{array}$$

Here, ${\varvec{W}}_{{{\varvec{output}}}} \in {\mathbb{R}}^{2 \times 2d} ,\user2{ b}_{{{\varvec{output}}}} \in {\mathbb{R}}^{2}$. Finally, ${\varvec{z}} = \left[ {y_{0} , y_{1} } \right]$ was inputted into the softmax layer and binary classification on whether the ligand is active or not was performed.

### Hyperparameter optimization and docking simulation settings

We used almost the same neural network structure and hyperparameters used in the previous method by Tsubaki et al. Thus, the hyperparameter values are the same as previous research except for the parameters related to protein features (i.e. *r*-radius of a protein pocket subgraph). The value was determined by using the training dataset. Details of hyperparameters used in the proposed method are shown in Table 6.

**Table 6:** Hyperparameters used in this research

Hyperparameter	Value/method
Dimensions of feature vector	10
Compound r-radius subgraphs	2
Layers of compound graph neural network	3
Protein pocket r-radius subgraphs	1
Layers of protein pocket graph neural network	3
Learning rate	0.001
Learning rate decay	0.5
Decay interval	10
Optimization function	Adam
Epoch size	100
Batch size	1

We performed experiments on target proteins for which protein–ligand complex structures were obtained. Therefore, we assumed that the central coordinates of a ligand were the central coordinates of the pocket. A 24 Å × 24 Å × 24 Å cube centered on that coordinates was defined as the search range of AutoDock Vina. In addition, we set *num_modes* to 100, *energy_range* to 3, *exhaustiveness* to 8.

## Data Availability

The source codes and datasets are available via http://www.cb.cs.titech.ac.jp/bmc2020/.

## Abbreviations

AUROC: Area under receiver operating characteristic
TPR: True positive rate
FPR: False positive rate
